# CAR-T Cells in the Treatment of Nervous System Tumors

**DOI:** 10.3390/cancers16162913

**Published:** 2024-08-22

**Authors:** Ugo Testa, Germana Castelli, Elvira Pelosi

**Affiliations:** Department of Oncology, Istituto Superiore di Sanità, Viale Regina Elena 299, 00161 Rome, Italy; germana.castelli@iss.it (G.C.); elvira.pelosi@iss.it (E.P.)

**Keywords:** immunotherapy, chimeric antigen receptor T cells, nervous system tumors, brain tumors, glioblastoma, diffuse midline glioma, neuroblastoma

## Abstract

**Simple Summary:**

This review explores the emerging area of the therapeutic use of chimeric antigen receptor (CAR)-T cells in nervous tissue tumors. Through a detailed analysis of the existing literature, this paper highlights the most recent applications of CAR-T cells to the therapy of pediatric and adult nervous system neoplasia. Furthermore, it discusses the potential mechanisms underlying sensitivity or resistance to CAR-T cell-based therapies. Overall, this review underscores the importance of CAR-T cell therapies to the treatment of some nervous system tumors.

**Abstract:**

Chimeric antigen receptor T cells (CAR-Ts) have shown a remarkable efficacy in hematological malignancies but limited responses in solid tumors. Among solid tumors, CAR-T cell therapy has been particularly explored in brain tumors. CAR-T cells have shown a limited clinical efficacy in various types of brain tumors due to several factors that have hampered their activity, including tumor antigen heterogeneity, the limited access of CAR-T cells to brain tumor cells, limited CAR-T cell trafficking and in vivo persistence and the presence of a highly immunosuppressive tumor microenvironment. Despite these considerations, some recent studies have shown promising antitumor activity of GD2-CAR-T cells on diffuse midline gliomas and neuroblastomas and of CARv3-TEAM-E cells in glioblastomas. However, strategies are required to improve the effect of CAR-T cells in brain tumors, including advanced CAR-T cell design with multiple antigenic targeting and incorporation of combination therapies.

## 1. Introduction

In the last few years, cancer treatment has explored new strategies targeting molecules specifically expressed on tumor cells. One of these approaches was based on immunotherapy which, through different strategies, promotes the host’s immune system to specifically kill cancer cells, sparing normal cells. Among the numerous different types of immunotherapies developed in the last two decades, one of growing interest is represented by the generation of T lymphocytes engineered to express chimeric antigen receptors (CARs). CAR-T cells represent a very powerful tool for promoting anti-cancer response based on two different mechanisms: promotion of the killing of tumor cells expressing a given antigen and induction of an immune response in the tumor microenvironment.

A fundamental property of CAR-T cells that supports their application to cancer therapy is represented by their capacity to promote recognition and killing of target cells in a major histocompatibility complex (MHC)-independent manner, thus bypassing a physiological mechanism required by normal T cells; the bypass of MHC restriction by CAR-T cells is an important property since MHC downregulation represents one of the key mechanisms of immune escape adopted by cancer cells [1].

**Figure 1:**
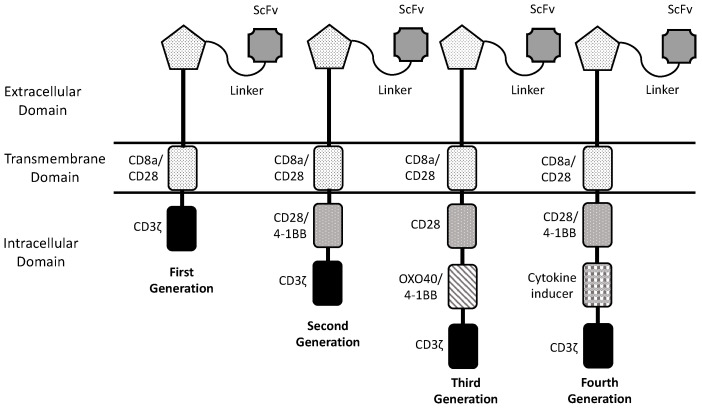
The structure of different CAR generations. The core structure of a CAR involving components of the extracellular domain, the transmembrane domain and the intracellular domain. The evolution of CAR structure involves the passage from first-generation CARs with only a signaling motif in the intracellular domain to second-generation CARs containing one co-stimulatory molecule, to third-generation CARs with two co-stimulatory molecules and to fourth-generation CARs with a cytokine inducer in addition to two co-stimulatory molecules.

The basic structure of a CAR-T in its simplest form is represented by an antibody or ligand-derived ectodomain (conferring the antigen-binding capacity) fused with a hinge, transmembrane domain and an intracellular T-cell-signaling domain. This simple structure corresponds to the first-generation CARs characterized by the presence of a single intracellular domain; however, the CAR-T cells engineered with first-generation CARs, although exhibiting an efficient antitumor potential, display in vivo a limited proliferation, survival and trafficking, thus limiting their antitumor efficacy [2]. (Figure 1) Given these limitations, two strategies have been adopted to improve CAR-T cell expansion and persistence consisting in the introduction of one or two co-stimulatory domains in the CAR structure, allowing the development of second-generation and third-generation CAR-T cells, respectively [2]. (Figure 1) Co-stimulatory domains commonly used for CAR-T cell generation are represented by CD28, 1-1BB, CD27 and ICOS [2]. Finally, more recently, CAR generation was further optimized through introduction of antitumor cytokines, such as interleukin-2, in the CAR structure, thus allowing the development of fourth-generation CAR-T cells [2] (Figure 1).

The process of autologous CAR-T cell manufacturing involves various steps which usually take 2 weeks and include initial cell isolation, T cell activation, transduction of CAR transgene, cell expansion, final formulation and product release testing [3,4]. Most CAR-T cell products are frozen, cryopreserved in liquid nitrogen and thawed at bedside prior to infusion into patients. Recently, abbreviated manufacturing procedures have been introduced, which are able to reduce the whole ex vivo process of manufacturing to 2–3 days [3]. 

CAR-T cells have been introduced in the clinical treatment of hematological malignancies, achieving impressive clinical results in relapsed/refractory B cell malignancies, including lymphoma, leukemia and myeloma [5,6]. Following remarkable clinical outcomes in hematological malignancies, the FDA approved six CAR-T cell products for indications such as lymphoma, leukemia and myeloma [5,6].

However, CAR-T cell applications in the treatment of solid tumors remain limited, due to several constraints: the limited or suboptimal access of CAR-T cells to solid tumor cells in organs, the presence of a highly immunosuppressive tumor microenvironment, limited CAR-T cell trafficking and in vivo persistence, T cell exhaustion, tumor antigen heterogeneity and lack of suitable target antigens [7,8].

In spite these limitations, a growing number of phase I/II clinical trials are exploring CAR-T cell therapy in solid tumors. Brain tumors represent the most common solid tumor types undergoing clinical trial evaluation for CAR-T cell safety and efficacy [7,8].

Most of the studies involving the study of CAR-T cells in tumors involve the use of monospecific CAR-T cells targeting a single tumor antigen, and this approach, particularly in solid tumors and in brain cancers, is limited by tumor antigen heterogeneity, antigen escape and a tumor-immunosuppressive microenvironment. An important strategy for bypassing these limitations consists of targeting multiple antigens simultaneously on the surface of tumor cells. This strategy is based on the use of bispecific or trispecific CAR-T cells that mitigate the negative impact of antigen heterogeneity and antigen loss. Several combinations of target antigens have been proposed, such as EGFRvIII and IL-13Rα2, and EGFRvIII and EGFR, with the main objective of offsetting immune escape in brain tumors. Preclinical studies have shown that CAR-T cells simultaneously targeting EGFRvIII and IL-13Rα2 display marked and durable antitumor activity in murine models of heterogeneous GBM [9]. The development of bispecific antibodies targeting HER2 or EGFR and CD3 offers new perspectives for immunotherapy in solid tumors, including brain tumors [10].

Natural killer (NK) cell-based immunotherapy displays several interesting properties suitable for the treatment of GBM and other brain tumors, including the absence of restriction via antigen–antibody reactions and good penetration into brain tumor tissue [11]. Particularly, genetically engineered NK cells, including CAR-NK cells adapter CAR-NK, appear to be particularly suitable for glioblastoma immunotherapy [11]. Preclinical studies have shown that CAR-NK cells retained the constitutive antitumor activity of NK cells and acquired the capacity to specifically kill tumor cells through antigen targeting [12]. These studies have shown significant therapeutic effects in GBM models and fewer adverse events compared to CAR-T cells [12]. In spite their potentialities, some challenges limit the efficacy CAR-NK cells in solid tumors, mainly represented by the short lifespan and rapid turnover in vivo compared to the longer-lived T lymphocytes [13]. 

Combination therapies represent an important approach to try to bypass the immunosuppressive microenvironment of brain tumors and to improve CAR-T cell efficacy and durability. In this context, various combinations of CAR-T cells with various agents, including antibodies, small molecules and viruses, have been proposed [14]. One of these associations is based on the combination of monoclonal antibodies targeting PD-1 or PD-L1 (immune checkpoint inhibitors, ICIs), to enhance the antitumor activity of tumor-infiltrating T lymphocytes, with CAR-T cells [14]. Thus, experimental studies have shown that Nivolumab (anti-PD-1 monoclonal antibody) potentiates the antitumor activity of GD2 CAR-T cells in orthoptic NOD/SCID GBM mouse models [15]. 

The efficacy of CAR-T cells may be potentiated by administration in association with oncolytic viruses. Oncolytic viruses (Ovs) are attenuated viruses grown in laboratories, exhibiting the property of selectively targeting and eradicating tumor cells [16]. Several ongoing clinical trials are evaluating the safety and the therapeutic potential of different Ovs in GBM treatment. Furthermore, some immunotherapy combinations with oncolytic viruses, particularly those including CAR-T cells and Ovs, are under development to both improve outcomes and to reduce adverse effects [17]. Examples of Ovs associated with CAR-T cells are given by Ovs expressing IL-15/il-15Rα, potentiating the antitumor effect of EGFR-CAR-NK cells in GBM mouse models [18], or Ovs expressing IL-7, improving the efficacy of B7H3-CAR-T cells for glioblastoma therapy [19].

The treatment of brain tumors with CAR-T cells show additional difficulties due to the semipermeable properties of the blood–brain barrier leading to difficult access to the tumor mass [20]. A fundamental concern of the current CAR-T cell therapy is that systemic delivery of CAR-T cells limits on-tumor, on-target efficiencies due to the obstacles raised by the blood–brain barrier and may require higher CAR-T cell doses to achieve the migration of a sufficient number of T cells at the level of brain tumor sites. To achieve the limitations related to the ineffective chemotaxis of CAR-T cells to disease sites, locoregional CAR-T cell delivery via infusion through an intracranial catheter can be used. CAR-T cell infusion to the brain includes three different ways of administration: delivery via the blood, delivery via cerebrospinal fluid and delivery in the tumor cavity (intracavitary) or in the brain ventricles (intraventricular). Surgically implanted Ommaya and Richman reservoir catheters, easily accessible with a small needle for repeated drug delivery of CAR-T cells in adults as well as in pediatric patients with CNS tumors [21]. Two additional techniques of CAR-T administration directly in the CNS are represented by spinal intrathecal infusion (SII) and disruption of the blood–brain barrier by focal ultrasound; SII is a technique well known in pediatric oncology used for intermittent infusion of chemotherapy [20]. Numerous trials exploring anti-CD19 CAR-T cells in hematologic malignancies have shown the capacity of engineered T cells to cross the blood–brain barrier [20]. However, studies in preclinical models have supported a superiority of locoregional (intracavitary or intraventricular) CAR-T cell infusion compared to systemic intravenous infusion, in terms of both enhanced antitumor efficacy and reduced toxicity [22].

In this review, we focus on recent studies based on CAR-T cell therapy in pediatric and adult brain tumors. The results of these studies, as well as the challenges of CAR-T cell therapy for brain cancers, are discussed and analyzed.

## 2. Experimental and Clinical Studies of CAR-T Cell Therapy in Brain Tumors

A consistent number of experimental and clinical studies have explored the targeting of brain tumors using CAR-T cells engineered to interact with surface antigens expressed on brain tumor cells. (Table 1)

### 2.1. HER-2

The EGFR family member HER-2, also known as ERBB2, is overexpressed in brain tumor cells and represents a potential tumor target of CAR-T cell therapy.

Glioblastoma (GBM) is the most common and aggressive malignant primary human brain cancer in adult patients, with a median overall survival from diagnosis of about 12–18 months. GBM is considered as one of the most aggressive and lethal among human malignancies and is resistant to every type of therapeutic strategy, including cytotoxic chemotherapy and molecular targeted therapies. Furthermore, GBMs are intrinsically resistant to T-cell-based immunotherapies, including those based on PD1/PDL1 immune checkpoint-blocking agents; thus, marked resistance to immunotherapy seems to be related to the paucity of T cells and to the immunosuppressive microenvironment present in these tumors, characterized by abnormal vascular niches and by the prominent infiltration of immunosuppressive macrophages.

Liu et al. have explored HER-2 expression in 43 primary GBM cell lines and observed significant positivity in 76% of cases; HER-2 expressed on glioblastoma cells is recognized by cytotoxic T cells [41]. Ramezani and coworkers have explored HER-2 expression by immunohistochemistry in 107 primary brain tumors and observed a higher frequency of positive tumors among high-grade astrocytomas (55%) than in low-grade astrocytomas (26%) [42]. Mineo and coworkers have compared the HER-2 expression in de novo glioblastoma (primary) and GBM resulting from anaplastic transformation of low-grade glioma (secondary) and observed in the former ones a high HER-2 expression and in the latter ones a lower HER-2 expression [43]. High HER-2 expression was associated with shorter survival [43].

A preclinical study showed in an orthoptic murine xenograft model the efficacy of HER-2-specific CAR-T cells generated from 10 GBM patients in mediating the killing of autologous GBM cells, including the fraction of CD133-positive cancer stem cells [44].

In a clinical setting, the first patient treated with HER-2-targeted CAR-T cell therapy succumbed to death due to a cytokine storm; this patient (colon cancer) received the treatment with a CAR-T engineered with ScFv from high-affinity Trastuzumab antibody and co-stimulatory domains CD28 and 41BB [45]. Given this severe event, a new CAR-T was redesigned including an ScFv from a low-affinity FRP5 antibody and a different co-stimulatory domain inducing lower cytokine release in vivo; a study was carried out using these CAR-T cells in patients with progressive HER-2-positive glioblastoma, involving one or more infusions (up to 1 × 10^8^ CAR-T cells), administered without prior lymphodepletion [23]. A total of 17 patients were enrolled, and 16 were evaluable for response: 1 had partial response for more than 9 months, 7 had stable disease for 8 weeks to 29 months and 8 progressed after T cell infusions. No dose-limiting toxic effects were reported [23]. The median OS post-treatment was 11.1 months and 24.5 months post-diagnosis; however, no conclusions can be drawn about a possible survival benefit [23].

Vitanza and coworkers have reported the preliminary results of the phase I BRAINChild-01 clinical trial involving locoregional infusion of HER-2-specific CAR-T cells in children and young patients with recurrent or refractory CNS tumors; the observations made in the first three treated patients showed no dose-limiting toxicities or evidence of local immune activation (with high concentrations of CXCL10 and CCL2 in the cerebrospinal fluid), thus supporting the clinical feasibility of this study [46].

Studies in an orthoptic xenograft model of HER-2^+^ breast cancer metastasis to the brain have shown an enhanced proliferative capacity and a reduced T cell exhaustion phenotype of HER-2-CARs containing the CD28 co-stimulatory domain following regional intraventricular delivery of HER-2 CAR-T cells for the treatment of multifocal brain metastases [47].

Thus, two phase I clinical trials (NCT03389230 and NCRT03696030) have started the evaluation of these optimized HER-2-specific CAR-T cells among patients with HER-2-positive malignant gliomas and HER-2-positive metastases of breast cancers; two other studies are evaluating locoregional treatment of recurrent and/or refractory pediatric CNS tumors (NCT03500991 and NCT02442297).

Recent preclinical studies have reported the generation of HER-2-targeting CAR-T cells exerting marked antitumor activity either through the development of third-generation CAR-T cells or through the development of CAR-T cells with low-affinity HER-2CARs [48]. Furthermore, this study also showed that peritumoral intravenous CAR-T cell administration resulted in better glioblastoma inhibition compared to intravenous administration [49].

Burger et al. have explored the safety and the efficacy of intravenous injection of NK cells engineered with HER-2-targeted CAR in patients with R/R GBM [50]. Particularly, in preclinical studies, NK-92/5.28.z cells displayed high and selective cytotoxicity against HER2-positive GBM cells, even under hypoxic conditions or an immunosuppressive microenvironment [51]. The phase I CAR2BRAIN study enrolled nine patients with R/R HER2-positive GBM treated with a single intracranial CAR-NK dose at a dose of 1 × 10^7^, 3 × 10^7^ or 1 × 10^8^ HER2-targeted CAR-NK cells [50]. The treatment was well tolerated and no toxicity-limiting disease or adverse neurologic events were observed [50]. The best responses were stable disease observed in five patients; two patients treated at 1 × 10^8^ dose both achieved a PFS of 37 weeks and an OS of 98 and 135 weeks, respectively [50].

### 2.2. EGFRvIII

Epidermal growth factor amplification is one of the most recurrent genetic alterations of glioblastoma and is present in more than 50% of primary glioblastomas and in less than 10% of GBMs evolving from recurrent low-grade gliomas; 30% to 50% of EGFR-amplified GBMs exhibit an in-frame deletion of exons 2 to 7, thus generating a truncated receptor with loss of the extracellular ligand-binding domain, called EGFR variant III (EGFRvIII); EGFRvIII is expressed only in tumor cells and is associated with constitutive receptor signaling. Given this tumor-specific expression, EGFRvIII represents a therapeutic target in a part of GBM patients, potentially suitable for CAR-T-mediated targeting.

The study of relevant genetic mouse models of GBMs identified distinct immune landscapes associated with expression of EGFR wild-type and mutant EGFRvIII; particularly, the accumulation of polymorphonuclear myeloid-derived suppressor cells (PMN-MDSC) was more pronounced in EGFRvIII-driven GBMs, in association with resistance to PD-1 checkpoint blockade immunotherapy [52].

O’ Rourke et al. have performed a first-in-human study of intravenous delivery of a single dose of autologous CAR-T cells targeting EGFRvIII in 10 recurrent GBM patients [24]. Manufacturing and infusion of EGFRvIII-directed CAR-T cells was feasible and well tolerated; no therapeutic responses were observed in these patients (only one patient had residual stable disease for over 18 months of follow-up); all patients displayed transient expansion of CART-EGFRvIII cells in peripheral blood, and imaging studies suggested trafficking of CAR-T cells at the level of regions of active GBM proliferation [24].

Goff et al. have investigated in a pilot phase I study the association of EGFRvIII-targeted CAR-T cell therapy with IL-2 infusion post-transfer, in R/R GBM patients [25]. Cell infusion products ranged from 6.3 × 10^6^ to 2.6 × 10^10^ anti-EGFRvIII CAR-T cells and were administered intravenously after lymphodepletion; median overall survival was 6.9 months, with two patients surviving over 1 year, and a third patient was alive at 59 months [25]. Two patients experienced severe hypoxia and one of these patients succumbed to death after CAR-T cell infusion at the highest dose [25]. No objective responses were observed in the treated patients [25].

In order to reduce the risk of a tumor recurrence after anti-EGFRvIII CAR-T cell therapy by tumor cells expressing wild-type EGFR protein, Choi et al. have developed a peculiar strategy based on the use of a bicistronic construct driving the expression of a CAR specific for EGFRvIII and a bispecific T cell engager (BiTE) against EGFR [53]. Autologous T cells engineered with CARvIII-TEAM-E were intraventricularly reinfused to three patients with recurrent GBM; none of these patients developed toxicities over grade 3 [26]. A dramatic radiographic tumor regression shortly after a single infusion of CARvIII-TEAM-E CAR-T cells was observed in all three patients, but this response was transient in two of them, which correlated with limited persistence of CARvIII-TEAM-E cells [26]. The presence of CAR-T cells in the peripheral blood peaked 3 weeks after CAR-T cell infusion. The early responses observed in this study will support future studies based on the intraventricular infusion of cell-based therapies to GBM patients. However, the transient response observed in two of the three treated patients underscores the absolute need for additional research to improve and prolong in the duration of efficacy of this therapy. Finally, this study supports the value of multitargeted CAR-T cell therapy in the treatment of brain tumors.

The study of the apheresis infusion products from the first trial of EGFRvIII-directed therapy showed that CAR-T cell therapy targeting EGFRvIII induced an upregulation of programmed death-ligand 1 (PD-L1) expression in the tumor microenvironment; furthermore, the expression of PD-1 in CAR-T infusion products correlated with clinical response (PFS) [54]. These observations have supported a phase I trial (NCT03726515) exploring the concomitant intravenous administration of CAR-T-EGFRvIII cells with anti-PD-1 monoclonal antibody Pembrolizumab in seven patients with newly diagnosed GBM [27]. No limiting toxicities were observed; mPFS was 5.2 months, and mOS was 11.8 months; no objective responses were observed in these patients [27]. A comparison of tumor microenvironments in tumor specimens obtained before and after CAR-T cell therapy showed a consistent evolution of the infiltrating myeloid and T cells, with more exhausted, regulatory and interferon-stimulated T cells at relapse [27].

Another recent study explored the safety and the efficacy of intracellular administration of bivalent CAR-T cells engineered to target both EGFR and interleukin-13 receptor alpha 2 (IL-13Rα2) in six recurrent GBM patients [28]. All six treated patients had progressive multifocal disease at the time of treatment. In all the six treated patients (three with 1 × 10^7^ cells and three with 2.5 × 10^7^ cells), infusions of CAR-T-EGFR-IL-13Rα2 cells were associated with early-onset neurotoxicity treated with dexamethasone and anti-IL1R antibody; radiologic evidence of tumor reduction was observed in all treated patients, but none reached criteria for an objective response [28]. A substantial CAR-T cell abundance and cytokine release was observed in all six treated patients [28]. The results of this study are still preliminary and need an evaluation after the completion of dose escalation and a longer follow-up of patients.

Recently, a novel GCT102 CAR-T was developed, engineered with EGFRvIII-specific ScFv, targeting EGFRvIII with high affinity; in a xenograft model of human GBM, GTC102 CAR-T cells efficiently killed tumor cells with decreased cytokine secretion [55]. In different preclinical GBM models, GTC102 CAR-T cells displayed specificity for tumor cells expressing EGFRvIII, thus supporting future studies aiming to evaluate their activity in clinical settings [55].

### 2.3. Interleukin-13 Receptor alpha2 (IL-13Rα2)

Two IL-13 receptor proteins were identified: (i) IL-13Rα1, a component of the signaling, heterodimeric high-affinity receptor for IL13- that is shared with IL-4; (ii) IL-13Rα2, a monomeric, IL-4-independent receptor, highly specific for IL-13 and not capable of binding IL-4. IL-13Rα1 and IL-13Rα2 are two members of the hematopoietic receptor superfamily exhibiting very low sequence homology. Human malignant glioma cells express high levels of IL-13Rα2, while this receptor chain is scarcely expressed on normal astrocytes [56]. IL-13Rα2 is a high-affinity receptor of the inflammatory cytokine IL-13 and its preferential expression in many solid tumors, including malignant gliomas, supports its value as a potential attractive target for cancer immunotherapy [57].

Brown et al. have reported a first-in-human pilot safety and feasibility trial evaluating CAR-engineered, autologous primary human CD8^+^ lymphocytes targeting IL-13Rα2; this CAR recognizes IL-13Rα2 via a membrane-tethered IL-13 ligand mutated at a single site, E13Y, to reduce binding to the IL-13Rα1/IL-4Rα complex and initiates cytolytic killing via an intracellular CD3ζ T-cell-activating domain [58]. In this pilot study, three patients with recurrent glioblastoma were treated with up to 12 local infusions (tumor intracavitary) of IL13(E13Y)-zetakine CD8^+^ CAR-T at a maximum dose of 10^8^ CAR-T cells [58]. A transient anti-glioma effect was observed in two of these three patients [58]. The treatment was well tolerated, with manageable transient brain inflammation [58]. In 2016, it was reported that a complete tumor regression observed in a patient with recurrent glioblastoma after treatment with IL-13Rα-CAR-T cells infused through two intracranial delivery routes (infusion into the resected tumor cavity, followed by infusions into the ventricular system [50]. No toxic event of grade 3 or higher was observed [59].

Based on the remarkable clinical response observed in this patient to IL13Rα2-CAR-T cells, it was hypothesized that additional mechanisms could be involved beyond the direct effect of CAR-T cells on tumor targets. Thus, it provided evidence that IL13Rα2 CAR-T cells exert effects on the endogenous immune system, mainly mediated through acute production of IFN-γ by CAR-T cells; IFN-γ mediates changes in the tumor microenvironment corresponding in an enrichment in activated memory or effector T cells in the lymphoid compartment and increases in activated myeloid cells and M1-type macrophages in the myeloid compartment [60]. These observations suggest a critical role for IFN-γ signaling in sustaining productive CAR-T cell therapy in GBM patients and show that CAR-T cells can activate endogenous T cell immunity, thus supporting a critical role for host innate and adaptive immunity for CAR-T cell therapy of GBM.

In 2024, Brown and colleagues reported the results of the phase I trial based on IL13(E13Y)-zetochine CAR-T cells involving the enrollment of 65 patients with recurrent high-grade gliomas (in large part, recurrent GBMs); this trial evaluated three routes of locoregional CAR-T cell administration (intramural (ICT), intraventricular (ICV) and dual (ICT and ICV) and two manufacturing platforms and a final arm of treatment based on ICT/ICV and an optimized manufacturing procedure [29]. All routes of CAR-T cell delivery and infusion dose levels (from 2 × 10^6^ to 200 × 10^6^ CAR-T cells) were generally well tolerated, with grade 3 toxicities or above observed in 35% of patients; no dose-limiting treatment toxicities were observed for any patient [29]. The recommended dose for phase II was estimated to correspond to 200 × 10^6^ CAR-T cells. A total of 58 patients were evaluable for response, and 50% of them displayed stable disease or better; 22% of patients achieved confirmed stable disease or better for ≥90 days, with 8/13 having relapsing high-grade glioma, grade 4; two patients achieved a partial response and one a complete response (all these three patients have IDH-mutated gliomas) [29]. A second patient achieved a complete response after additional CAR-T cell cycles off protocol. For GBM patients, mOS was 7.7 months for all patients and 10.2 months for those with ICT/ICV and optimal CAR-T cell manufacturing [29]. CAR-T cell administration was associated with increased levels of inflammatory CNS cytokines; pre-treatment intratumoral CD3 levels were positively associated with patients’ survival [29].

Brown and coworkers have performed a first-in-human study of locally administered, glocorticoid-resistant, allogeneic CAR-T cells administered to six patients with recurrent GBM under treatment with dexamethasone to attenuate tumor-related neuro-edema [61]. Zinc finger nuclease-directed disruption of the glucocorticoid receptor gene was used for the generation of dexamethasone-resistant IL-13Rα2-targeted CAR-T cells; these dexamethasone-resistant cells retained effector function in the presence of dexamethasone, without any rejection of the therapeutic allogeneic cells [61]. The treatment was well tolerated and showed in four of the six treated patients transient antitumor effects [61].

Stern and coworkers have identified two IL-13 variants (C4 and D7), bearing mutations that decrease binding affinity for IL-13Rα1 but did not markedly change affinity for IL-13R α2; in vivo biodistribution of CAR-T cells bearing these IL-13 variants were better able to traffic away from IL-13Rα1-positive lung tissue [62]. This study supports the use of CAR-T cells with IL-13 mutants selective for IL-13Rα2. In line with these observations, Kim et al. have reported the development of CAR-T cells with modified IL-13 (IL-13 was modified on the extracellular domain by substitution of amino acids with E13K, R66D, S69D and R109K) preferentially recognizing IL-13Rα2 and not IL-13Rα1 on malignant glioma cells; YYB-103 CAR-T cells exhibited selectivity for IL-13Rα2-positive tumor cells, and their intravenous infusion elicited inhibition of tumor growth in orthoptic models of human glioblastoma [63]. Furthermore, in a recent study, Loland and coworkers developed novel CAR-T cells with an ScFv clone exhibiting high affinity for IL-13Rα2 and high antitumor activity in models of human gliomas [64].

### 2.4. Disialogangloside (GD2)

GD2 is a carbohydrate-containing sphingolipid composed by a ceramide with two sialic residues attached via three monosaccharide links. The intracellular synthesis of GD2 occurs in the Golgi apparatus through the action of two different glycosyltransferases. GD2 is a membrane tumor-associated antigen expressed by a wide range of tumors of neuroectodermal and epithelial origin, such as neuroblastoma, glioma, retinoblastoma, medulloblastoma, melanoma, small-cell lung cancer and other tumors. GD2 expression can be detected also on normal central and peripheral nervous system cells but its expression is markedly higher on tumor cells. Given these properties, GD2 is a potentially attractive target for immunotherapy of brain cancers.

Pediatric-type diffuse high-grade gliomas comprise four types of aggressive gliomas: diffuse midline glioma, H3K27 altered; hemispheric glioma, H3 G34 mutant; diffuse pediatric-type high-grade glioma, H3 and IDH wild-type; and infant-type hemispheric glioma. Diffuse midline glioma (DMG), H3K37 altered and diffuse hemispheric glioma, H3G4 mutant, are characterized by point mutations in histones, causing widespread epigenetic alterations [65]. DMG with H3K27 alterations grow in CNS midline structures and are associated with poor outcomes [65]. Some studies explored the targeting of GD2 in diffuse intrinsic pontine glioma (DIPG) and other diffuse midline gliomas (DMGs) with mutated H3KM27M, characterized by a high, uniform GD2 expression [66]. Anti-GD2 CAR-T cells incorporating a 4-1BB co-stimulatory domain showed killing of DMG cells in vitro and high antigen-dependent cytokine generation [66]. Furthermore, in patient-derived H3-K27M^+^ DMG orthoptic xenograft models, systemic administration of anti-GD2 CAR-T cells cleared engrafted tumor cells expressing low levels of GD2 [66].

This preclinical study has supported the development of a phase I clinical study (NCT 04196413) evaluating intravenous infusion of autologous anti-GD2 CAR-T cells (1 × 10^6^ GD2 CAR-T cells per kg), followed by optional repeated intracerebroventricular (ICV) infusion of these CAR-T cells, in pediatric and young adult H3K27M-mutant DMG patients [30]. Radiographic and clinical benefit was observed in three out four patients, including a pronounced tumor reduction and neurologic improvement; the one nonresponsive patient displayed elevated levels of immunosuppressive cytokines in cerebrospinal fluid, such as transforming growth factor-beta (TGF-β) [30]. Toxicity was largely related to the location of the tumor and was reversible with intensive supportive care; off-target toxicity was not observed. It is important to note that the addition of ICV dosing improved or stabilized clinical responses and was associated with increased levels of immunosuppressive myeloid cells in the CSF compared to IV infusions [30]. These initial observations hold promise for additional clinical trials in DMG patients involving the administration of GD2-CAR-T cells. In a second study presented at AACR 2022, the results of the first 13 patients enrolled in this study were reported: 4 treated at 1 × 10^6^ GD2-CAR-T cells/Kg and 9 treated at 3 × 10^6^ GD2-CAR-T cells/Kg [31]. Patients responded to the initial IV infusion of GD2-CAR-T cells ICV GD2-CAR-T cells every 4–8 weeks for a maximum of 12 doses [31]. Concerning safety, three patients treated at 3 × 10^6^ CAR-T cells/Kg experienced grade 4 cytokine release syndrome (CRS); furthermore, all treated patients displayed transient tumor inflammation-associated neurotoxicity [31]. Of the patients, 9/10 adequately assessed for benefit displayed radiographic and/or clinical benefit after CAR-T cell IV infusion; one participant, a 31-year-old with sDMG, experienced a near-complete (>95%) reduction in tumor volume and a 17-year-old with DIPG experienced a near-complete (>98%) reduction in volume of a pontine tumor; ICV infusion was not associated with high-grade CRS; four subjects confirmed to receive ICV infusions in the study and continued to display radiographic benefit at +11, +9.5, +8 and +7 months after enrollment (importantly, two of these four patients displayed a near-complete reduction (95–98%) in tumor volume) [31]. A more mature assessment of these patients showed that (i) 1 × 10^5^ GD2-CAR-T cells/kg by IV is the maximum tolerated dose; (ii) four patients displayed major tumor volumetric reductions (52%, 54%, 1% and 100%); (iii) one patient showed a complete response ongoing for >30 months since enrollment; and (iv) eight patients displayed a clear neurological benefit as evidenced by an improvement in neurological deficits [67]. The 13 patients with DMG included in this study were explored for immunity-related changes observed during treatment with GD2-CAR-T cells [57]. GD2-CAR-T cell expansion following IV infusion was observed at the level of peripheral blood and persisted during ICV infusions but decreased over time; GD2-CAR-T cell expansion was observed also at the level of CSF after multiple repeated infusions [68]. Increased cytokine chemokine levels (such as IL-6 and IFN-γ) were present in the peripheral blood following IV GD2-CAR-T cell infusions, whereas chemokine/cytokine levels (such as CCL2 and CXCL9) were more pronounced in CSF following ICV CAR-T cell infusions [68]. Single cell analysis of CSF cells showed, after IV CAR-T cell infusion, an increase in CSF of regulatory T cells and suppressive myeloid cell populations compared to the baseline; these immune suppressive cells were reduced following ICV infusions [68]. Given the positive results observed in these patients, two new arms of this study were launched to better assess safety and the activity and to define the recommended phase II dose for ICV delivery of GD2-CAR-T cells without or with lymphodepletion. Furthermore, two additional phase I clinical trials (NCT 04099717, ACTRN 1262000675729) are evaluating DMG and DIPG patients as well as patients with other CNS tumors.

A recent phase I study explored the activity of GD2-CAR-T cells augmented with constitutive IL-7 receptor for treatment of high-grade pediatric gliomas [32]. The study originated from the observation that CAR-T cell efficacy for brain tumors is constrained by the immunosuppressive microenvironment present in these tumors and characterized also by the limited availability of immune-stimulatory cytokines; to overcome this hostile microenvironment, an engineered IL-7 receptor (IL-/R) was developed, promoting constitutive signaling through the Stat5 pathway and enhancing CAR-T cell survival, proliferation and function in murine and in vitro models [69]. Thus, to enhance T cell activity against GD2-positive brain tumors, Lin and coworkers have modified GHD2-directed CAR-T cells by introducing a constitutively active IL-7R [32]. Using these novel CAR-T cells, 11 pediatric patients (4–18 years) with DMG H3K27M-mutated or other recurrent GD2-expressing CNS tumors have been enrolled in a phase I study (NCT 04099797): 3 patients received treatment with GD2-CAR-T cells and 8 patients received treatment with CR7-GD2-CAR-T cells at two doses (1 × 10^7^ cells/m^2^; 3 × 10^7^/m^2^); all patients received standard chemo-radiotherapy before CAR-T cell infusions and lymphodepletion with fludarabine and cyclophosphamide before CAR-T cell infusions [67]. The CR7-GD2-CAR-T cell cohort developed grade 1 tumor inflammation-associated neurotoxicity in 88% of cases; CRS of grade 1 was observed in 75% of cases treated with CR7-GS2-CAR-T cells [32]. Most of the enrolled patients had a diagnosis of DNG H3K27M-mutated, and two patients had recurrent medulloblastoma. The three patients treated with GD2-CAR-T cells did not display any response; patients receiving CR7-GD2-CAR-T cells exhibited a transient improvement in baseline neurologic deficits: 2/8 had a partial response and 7/8 remained eligible for additional treatment cycles [32].

Only one clinical study explored the safety and the efficacy of GD2-directed CAR-T cells in GBM patients. This study evaluated 4SCAR-GD2 CAR-T cells in eight glioblastoma patients; 4SCAR-GD2 CAR-T cells were obtained through transfection of T cells with 4SCAR-GD2 lentiviral vector constructed with DNA sequences of GD2 scFv, CD28 transmembrane and cytoplasmic domains, co-stimulatory 4-1BB intracellular TRAF-binding domain, the CD3z chain intracellular domain and an inducible suicide caspase 9 gene [33]. Eight patients with diagnoses of relapsed GBM received the intravenous infusion of 2.5 × 10^6^ 4SCAR-GD2 T cells per Kg of body weight after lymphodepletion with fludarabine and cyclophosphamide; three of these patients with evidence of progressive disease and indication for surgery also received an intracavitary infusion of 1 × 10^5^ CAR-T 4SCAR-GD2 T cells per Kg of body weight [33]. The CAR-T cell treatment was well tolerated; 4SCAR-T cells expanded in vivo for 1–3 weeks and persisted at low frequency in peripheral blood; four patients displayed a partial response, lasting for 3 to 24 months; and three patients had progressive disease and one stable disease [33]. Immunohistochemical studies showed that 4SCAR-T cell infusions induced partial antigen loss and immune cell activation at the level of the tumor microenvironment [33].

Preclinical studies have shown the feasibility of efficient generation of GD2-targeting from the autologous lymphocytes of GBM patients endowed with a powerful and specific antitumor response against matched primary GBM cells [70]. Studies in orthoptic xenograft models of human GBM have shown significantly enhanced antitumor activity when GD2-targeting CAR-T cells were administered in association with Nivolumab [71] or using CAR-T cells manufactured with a retroviral vector encoding an interleukin-15 transgene alongside the GD2-specifric CAR [15].

GD2 is highly expressed in neuroblastomas, and the treatment of high-risk neuroblastomas with anti-GD2 antibody, G_-CSF, IL-2 and isotretinoin improves overall survival compared to standard treatment with isotretinoin [72]. A first clinical study (NCT 02761915) evaluated the response of 12 children with R/R neuroblastoma to treatment with escalating doses of second-generation GD2-CAR-T cells; no radiological responses were observed, and only three patients displayed regression of soft tissues and bone marrow disease [73]. Del Bufalo and coworkers reported the evaluation of GD2-CART01 cells in relapsed or refractory high-risk neuroblastoma patients [34]. In an academic, phase I/II study, 27 children with relapsed or refractory (12 refractory, 14 relapsed and 1 in complete response), highly pre-treated high-risk neuroblastoma were treated with autologous, third-generation GD2-CAR-T cells, expressing the inducible caspase 9 suicide gene [34]. Three dose levels were tested in phase I (3, 6 and 10 × 10^6^ cells/Kg of body weight); cytokine release syndrome occurred in 74% of patients and was mild in 20/21 patients; only one patient required activation of the suicide gene to obtain control of toxic events [63]. Six weeks after infusion of GD2-CAR-T01 cells, 33% of patients achieved a complete response and with a median follow-up of 1.7 years, a complete response was maintained in five of nine of these responding patients; 30% of patients had a partial response, 19% had stable disease and 19% were resistant to treatment [34]. Patients with a low disease burden had significantly lower survival than those with high disease burden: at 3 years, overall survival was 67% vs. 0%, respectively [65]. A total of 11 patients received additional infusions of GD2-CAR-T cells, with 3 complete responses (2 consolidations and 1 complete response in a patient relapsing after first treatment) and 3 partial responses [34].

A second study reported the response of 10 patients with R/R neuroblastoma with progressive disease to 4SCAR-GD2 T cells; after CAR-T cell treatment, 6 patients displayed stable disease at 6 months and 4 of them remained with stable disease at 1 year and alive after 3–4 years of follow-up; the median OS was 25 months [74].

A third study evaluated the safety and the antitumor activity of GD2-NKT cells in neuroblastoma patients. Natural killer T cells (NKT) are a rare subset of T lymphocytes that co-express TCRα/β and several markers associated with NK cells. NKT cells differ from conventional T cells, in that their TCRs are much more limited in diversity (invariant NKT cells). Vα24-invariant NKT cells have antitumor properties that can be enhanced by CARs. Thus, Heczey et al. have performed a first-in-human phase I trial evaluating autologous NKT cells co-expressing a GD2-specific CAR with IL-15 (GD2-CAR15-NKT) in 12 children with neuroblastoma [35]. A first initial report on this study assessed the feasibility of GD2-CAR15-NKT cell administration to three patients with neuroblastoma treated at dose level 1 (3 × 10^6^ cells/m^2^), showing good safety and promising antitumor activity [75]. This initial study was then extended to 12 patients at four dose levels: 3 × 10^6^, 1 × 10^7^, 3 × 10^7^ and 1 × 10^8^ cells/m^2^; 8 patients received a single dose, and 4 patients received two infusions [35]. The treatment was well tolerated, and no dose-limiting toxicities were observed; one patient displayed grade 2 CRS [35]. After the first infusion of GD2-CAR15-NKT cells, five patients had PD, four had SD and three had a PR; after the second infusion of GD2-CAR15-NKT cells, two patients had PD, one had PR and one had CR, maintained for 12 months [30]. Some markers correlated with response in these patients: the frequency of CD62L^+^ NKTs in CAR-T cell products was higher in responders; hyperexpression of BTG1 (antiproliferation factor 1) correlated with hyporesponsiveness of GD2-CAR15-NKT cells [35]. A preclinical study explored the efficacy of GD2-CAR-NK-92 cells for the treatment of DIPG; GD2-CAR-NK-92 cells displayed a strong cytotoxicity against DIPGs cells with high GD2 expression, associated with a good safety profile [51].

Very interestingly, the results of long-term survival of the first patients treated with GD2-CAR-T cells were recently reported. In 2008, Pule and coworkers engineered Epstein–Barr virus (EBV)-specific CTLs to express a CAR directed to GD2. In individuals with neuroblastoma, EBV-specific CTLs expressing a GD2-CAR survive better than T lymphocytes activated with anti-CD3 monoclonal antibody expressing a GD2-CAR but lacking virus specificity [36]. Infusion of these CAR-T cells was safe and was associated with tumor regression or necrosis in half of the cases [36]. Two reports have evaluated the long-term clinical and immunological consequences of these GD2-CAR-T cells of the first generation in 19 neuroblastoma patients: 8 in remission at infusion time and 11 with active disease at infusion; 6 weeks after infusion, the 8 patients in remission remained in remission, while the 11 patients with active disease displayed the following: 3 had PD, 2 had SD, 1 had PR, 3 had CR, and 2 had tumor necrosis; after a follow-up of 3–4 years, 4 patients in remission at infusion remained in remission, while 4 relapsed, and 2 CR and 9 relapsing patients were observed among patients with active disease at infusion time [37]. A recent study reported the final results of this study with a follow-up of 13–18 years [38]. Of the 11 patients with active disease at time of infusion, 3 patients had CR and one had PR; of the 3 patients with CR, 1 subsequently relapsed and 2 had sustained responses, 1 for 8 years and the other for 18 years [38]. Of the eight patients in remission at the time of infusion, five were disease-free at the time of the last follow-up ranging between 13 and 14 years after infusion [38]. Of the 19 patients, 12 died between 2 months and 7 years post-infusion, all due to relapsed neuroblastoma. The long-term results of this study are consistent with the observations made by Del Bufalo et al. [34] that patients with low tumor burden had significantly longer survival than those with a higher disease burden following GD2-CAR-T cell therapy.

A recent study showed that GD2 was expressed in a part of medulloblastomas, particularly in SHH (sonic hedgehog) and non-WNT/non-SHH group 4 subtypes [76]. Ciccone and coworkers have explored GD2 expression in a group of 52 medulloblastoma patients and reported GD2 positivity in 82 of the analyzed samples, with a marked interspecimen heterogeneity, with an average of 50 ± 36% of GD2-positive cells; the molecular medulloblastoma subtypes that were more positive were SHH, G4 and G3, while the WNT subtype was less GD2-positive [77]. The sensitivity of medulloblastoma cells to GD2-CAR-T cells identical to those used in the study of neuroblastoma patients [34] was explored, showing high levels of cell killing [77]. Importantly, the EZH2 inhibitor Tazemetostat induced a clear increase in GD2 expression on the surface of medulloblastoma cells, associated with a concomitant increase in their sensitivity to the cytotoxic effects of GD2-CAR-T cells [77]. GD2-CAR-T cells exerted a marked in vivo antitumor effect in xenograft medulloblastoma models, potentiated by pre-treatment with Tazemetostat; intravenously injected GD2-CAR-T cells were able to infiltrate medulloblastoma tissue [77]. Finally, the suicide gene present in the CAR vector was able, when drug-activated, to promote GD2-CAR-T cell eradication [77]. These preclinical studies have supported the development of a phase I/II clinical study (NCT 05298995) for the evaluation of the safety and therapeutic efficacy of GD2-CAR-T cells in high-risk medulloblastoma patients [77].

### 2.5. B7-H3

B7-H3 (CD276) is an immunomodulatory protein that has emerged as an attractive target for cancer immunotherapy for its low expression in normal tissues but high expression in many solid tumors. Particularly, B7-H3 is highly expressed in pediatric solid tumors, including neuroblastoma and in many brain tumors, including medulloblastoma, high-grade glioma, DMG, ependymoma and atypical teratoid rhabdoid tumor (ATRT); importantly, in these tumors, B7-H3 expression is high and comparable for its level of expression to that observed for GD2 [78]. B7-H3-targeted CAR-T cells display potent antitumor activity in patient-derived orthoptic xenografts of pediatric brain tumors [78].

B7-H3 mRNA and protein are also overexpressed in glioblastoma relative to normal brains in all GBM subtypes; particularly, high expression of B7-H3 was observed in >75% of glioblastomas [79]. Furthermore, in preclinical studies, B7-H3 appeared to be a suitable CAR-T target for glioblastoma [79,80].

A recent study reported the results of the first-in-human clinical study (Brain Child-03) and includes three distinct arms: arm A, including localized recurrent/refractory CNS tumors; arm B, including metastatic recurrent/refractory CNS tumors; arm C, including patients with DIPG enrolled at any time after radiotherapy. A first report concerned an interim analysis in the context of arm C [81]. The trial involved repeated weekly fixed doses (1 × 10^7^ cells) of intraventricular infusions of B7-H3 CAR-T cells to three patients with recurrent DIPG. The three DIPG patients of this study received 10, 12 or 18 infusions of B7-H3-CAR-T cells; two patients showed no response to treatment and one patient displayed a partial response with improvement in neurologic deficits; two patients showed an extended survival [81]. Patients exhibited correlative evidence of local immune activation and persistence of B7-H3-CAR-T cells in cerebrospinal fluid [72]. A more extensive report of the arm C of BrainChild study 03 was recently presented at the International Symposium on Pediatric Neuro Oncology (Philadelphia, 2024), including 21 patients with DIPG treated with multiple intraventricular infusions of B7-H3 CAR-T cells; these patients received a median of seven doses of CAR-T cells [81]. A total of 11 patients were enrolled after progression and survived 9.7 months after initial CAR-T cell infusions; 10 patients were enrolled before progression and survived 16.9 months; 3 patients were alive 3 years from diagnosis, and 2 of them are still on protocol therapy [39]. The most common adverse events were headache, nausea/vomiting, and fever [39].

A preclinical study supported the potential therapeutic value of B7-H3 targeting in atypical teratoid/rhabdoid tumors (ATRT), typically occurring in the CNS of children under 3 years of age [82]. ATRTs highly express B7-H3 and their targeting by B7-H3-CAR-T cells in ATRT xenograft cerebral models resulted in consistent antitumor activity [82]. These observations strongly support the development of clinical studies implying the targeting of ATRT with B7-H3 CAR-T cells [82].

Few studies have explored the safety and the antitumor activity of B7-H3 CAR-T cells in patients with glioblastoma and other high-grade gliomas. Tang et al. reported the case of a 56-year-old woman with recurrent GBM treated with multiple intratumoral infusions of B7-H3 CAR-T cells and achieving a dramatic antitumor response lasting up to the first five cycles of CAR-T cell infusions; unfortunately, after these first five cycles, the patient displayed tumor recurrence [83]. MAXIMUM Pharmaceuticals reported the results of the first seven patients with high-grade glioma (five GBM and two DMG) treated with multiple intralumbar injections of 2 × 10^7^ B7-H3-specific allogeneic universal CAR-T cells (B7H3 UCAR-T) [84]. The infusion of B7H3 UCAR-T cells was not associated with any toxic effect of grade 3 or higher and resulted in a significantly longer overall survival and a higher objective response rate than past data [84]. Zhang et al. reported the results of an additional phase I trial (NCT 05241392) involving 13 R/R glioblastoma patients treated with B7-H3-CAR-T cells (TX103), at three different dosage levels: 3 pts at 3 × 10^7^, 4 pts at 6 × 10^7^ and 6 pts at 15 × 10^7^ cells [40]. TX103 cells were administered biweekly, with five cycles as one course, intracavitary and/or intraventricularly through an Ommaya reservoir [40]. No dose-limiting toxicities were observed; some patients had grade 2 CRS; two patients displayed grade 3 neurologic events. At 12 months of follow-up, 83% of the patients survived; mOS was 20.3 months; two of the three patients from the dose 2 level achieved a PR and a CR, respectively [40].

## 3. Conclusions and Future Perspectives

Studies carried out in these last few years with monospecific CAR-T cells targeting antigens specifically or preferentially expressed in tumor brain cells, such as tumor-specific mutation of EGFR (EGFRvIII) or GD2 or IL-13Rα2, B7-H3 have shown the capacity to target antigen-expressing tumor cells. However, CAR-T cells against single antigens are hampered by tumor brain cell heterogeneity, leading to immune escape and progression in the majority of patients. These effects may be in part mitigated using CAR-T cells engineered with multitargeting capacity, as supported by the promising observations made by a pilot study using CAR-T cells targeting both ERGFRvIII and IL-13Rα2 in glioblastoma patients with recurrent disease. Similarly, the preliminary results of the INCIPIENT clinical trial based on CARv3-TEAM-ET cells engineered to target both EGFRvIII and wild-type EGFR showed a good safety profile and remarkable response in three R/R GBM patients. The results obtained in these studies need to be confirmed with additional patients and longer follow-up time.

The study of some pediatric tumors, such as relapsing/refractory neuroblastoma, showed good sensitivity to GD2-directed CAR-T cells, with a good potential therapeutic activity limited to patients with low tumor burden.

Furthermore, the study of GD2-specific and B7-H3-specific CAR-T cells in pediatric MDSs showed promising therapeutic activity limited to a minority of patients. At the moment, valuable strategies aiming to extend these responses to more patients remain undefined.

It is important that these studies also showed that locoregional CAR-T cell administration achieved better results compared to intravenous infusion.

Future perspectives will be based also on innovative therapeutic strategies, such as blood–brain barrier opening with low-intensity-pulse focused ultrasound and SynNotch CAR-T cell therapy. MR-guided focused ultrasound (MTgFUS) is an emerging technology allowing the transient and accurate permeabilization of the blood–brain barrier for targeted delivery to the central nervous system [85]. The procedure was well tolerated, with no adverse clinical and radiological events related to the procedure in patients with primary brain tumors and showed a transient opening of selected areas of BBB, making the procedure suitable for drug delivery [85]. Furthermore, this technique was shown to be suitable for therapeutic delivery of cells to the brain [86].

The study of chimeric Notch (synNotch), a type-1 transmembrane protein, has allowed for the generation of molecular platforms suitable for creating cell–cell contact signaling pathways, such as SynNotch CAR [87]. Choe and coworkers have used the SynNotch CAR system for the engineering of innovative CAR-T cells, based on a “prime-and-kill” strategy [87]. In this system, the initial antigen exclusively expressed on GBM cells (such as EGFRvIII) primes the T cells to induce the expression of CAR-targeting antigens, such as IL-13Rα2 or EphA2, thus eliminating tumor cells expressing IL-13Rα2 or EphA2 [87]. Importantly, this system allows the sparing of IL-13Rα2 or EphA2 targeting outside the CNS and favors the generation of CAR-T cells with a stem/naïve cell state, associated with prolonged in vivo persistence [87].

## Figures and Tables

**Table 1:** Main clinical trials involving CAR-T cells in adult and pediatric nervous tissue tumors.

Author	Trial and Phase	Target Antigen	Dose and Route of Administration	Number of Patients (Age)	Clinical Results	Adverse Events
Ahmed et al. 2017 [23]	NCT 01109059 Phase I	HER2	1 × 10^6^ to 1 × 10^8^ Intravenous without prior lymphodepletion	Pediatric 7 (10–17 yr) Adult 10 (30–69 yr) Recurrent GBM	1 partial response; 7 stable disease; 8 disease progression In adult pts: mOS 9.4 mo	No severe adverse events related to treatment
O’Rourke et al. 2017 [24]	NCT 02209376 Phase I	EGFRvIII	1.75 × 10^8^ to 5 × 10^8^ Intravenous after lymphodepletion	11 Adult recurrent GBM	1 SD; 10 no response Evidence of CAR-T cell trafficking to the tumor Reduction in target antigen	No off-tumor toxicity No CRS
Goff et al. 2019 [25]	NCT 01454596 Phase I	EGFRvIII	4 pts 1 × 10^7^; 3 pts 1 × 10^8^ 5 pts 1 × 10^9^; 5 pts 1 × 10^10^ Intravenous after lymphodepletion	18 Adult recurrent GBM	No objective response mPFS: 1.2 mo mOS: 6.9 mo	Severe adverse events and dose-limiting toxicities in the group at 1 × 10^10^ CAR-T cells
Choi et al. 2024 [26]	NCT 05660369 Phase I/Pilot	EGFRvIII	Patient 1: 2 infusions of 10 × 10^6^ CAR-T cells Patients 2 and 3: 1 infusion of 10×10^6^ CAR-T cells	3 Adult recurrent GBM	All patients displayed rapid and dramatic radiographic tumor regression. In 2/3 pts, this response was transient	No dose-limiting toxicity No adverse events greater than 3
Bagley et al. 2024 [27]	NCT 03726515 Phase I	EGFRvIII	4.65 × 10^7^ to 2 × 10^8^ 1–3 cycles of CAR-T cells 1 cycle of Pembrolizumab	7 (56–76 yr) Adult recurrent GBM	No objective responses mPFS: 5.2 mo mOD: 11.8 mo	No dose-limiting toxicity
Bagley et al. 2024 [28]	NCT 05168423 Phase I	EGFRvIII—IL-13Rα2 (bicistronic lentiviral vector)	3 pts (1 × 10^7^ cells/m^2^) 3 pts (2.5 × 10^7^ cells/m^2^) Intrathecal	6 (33–71 yr) Adult recurrent GBM	Significant reduction in tumor size at MRI, but none with radiographic objective response	Low-grade CRS Early moderate–severe neurotoxicity
Brown et al. 2024 [29]	NCT 02208362 Phase I	IL-13Rα2	57 pts From 2 to 200 × 10^6^ IL13-CAR-T cells ICT or ICV or ICT and ICV infusions	57 pts (16–71 yr) 41 pts GBM 2 pts DMG 7 pts gr4 Astrocytoma 7 pts gr3 Glioma	SD: 50% PR: 2 pts CR: 2 pts mOS: 7.7 mo GBM(all) mOS: 10.2 mo GBM(ICT and ICV)	No dose-limiting toxicity 2 pts grade3 neurologic events
Majzner et al. 2022 [30]	NCT 04196413 Phase I	GD2	IV (1 × 10^6^ cells/Kg) Optional ICV infusions in responding patients	4 (5–25 yr) DMG H3K2M7-mutated	75% patients clinical and radiographic response	Manageable toxicity On target, off-tumor toxicity not observed
Majzner et al. 2022 [31]	NCT 04196413 Phase I	GD2	4 pts IV (1 × 10^6^ cells/Kg) 9 pts IV (3 × 10^6^ cells/Kg)	13 (2–30 yr) DMG H3K2M7-mutated	90% pts clinical and radiographic response. 2 pts with complete response.	Grade 4 CRS in 3 pts at 3 × 10^6^ cells/Kg. Transient tumor inflammation neurotoxicity
Lin et al. 2024 [32]	NCT 04099797	GD2—IL-7R	3 pts: IV GD2-CAR-T cells 1 × 10^7^ cells/m^2^ Ppts: IV CR7-GD2-CAR-T cells 1 × 10^7^ cells/m^2^	11 (4–18 yr) 8 DMG H3K27M-mut 2 recurrent MB 1 ATRT (atypical teratoid rhabdoid tumor)	3 pts: GD2-CAR-T group: PD 8 pts CR7-GD2-CAR-T group: 2 pts PR; 6 pts SD	CRS grade 1 75% Tumor inflammation-associated toxicity grade 1 88%
Liu et al. 2023 [33]	NCT 03170141 Phase I	GD2	8 pts IV: 3 × 10^7^–2.1 × 10^8^ 3 pts IC: 2.6 × 10^6^–6.4 × 10^6^ 4SCAR-T cells	8 (3–63 yr) Recurrent GBM	3 pts: PD; 4 pts: PR; 1 pt: SD mOS from diagnosis: 19.7 mo; mOS from infusions: 11.5 mo	Grade 2 or 3 neurologic events: 2 pts
Del Bufalo et al. 2023 [34]	NCT 03373097 Phase I/II	GD2	17 pts single infusion; 11 pts multiple infusions 3 × 10^6^, 6 × 10^6^, 10 × 10^6^ GD2-CART01 cells/Kg	27 (2.7–18.6 yr) Refractory/relapsed Neuroblastoma	CR: 33%; PR: 30%; SD: 19%; NR: 19% At 3 yrs: OS 67% LDB vs. 0% HDB; EFS 58% LDB vs. 0% HDB	No dose-limiting toxicities Grade 1–2 CRS: 70% Grade 3 CRS: 4% Neurologic toxicity grade 1–2: 22%
Heczey et al. 2023 [35]	NCT 03294954 Phase I	GD2	3 pts 3 × 10^6^, 3 pts 1 × 10^7^, 3pta 3 × 10^7^, 3 pts 1 × 10^8^ GD2-CAR-NKT cells/m^2^ 8 pts single infusion, 4 pts double infusion	12 (2–12 yr) Refractory/relapsed Neuroblastoma	After first infusion: PR: 3pta; SD: 4 pts; PD: 5 pts After second infusion: CR: 1 pt; PR: 1 pt; PD: 2 pts.	
Pule et al. 2008 Louise et al. 2011 Che-Hsing et al. 2024 [36,37,38]	NCT 00085930 Phase I	GD2	19 pts IV infusion 1.2 × 10^7^ cells/m^2^ 5 × 10^7^ cells/m^2^ 3.1 × 10^8^ cells/m^2^	19 pts with R/R Neuroblastoma 11 with active disease 8 in remission	After a follow-up of 8–14 yrs survived: 5/8 in remission 2/11 with active disease	No dose-limiting toxicities
Brown et al. 2024 [29]	NCT 02208362 Phase I	IL-13Rα2	From 2 × 10^6^ to 200 × 10^6^ IL13-CAR-T cells ICT or ICV or ICT and ICV infusions	57 pts 41 pts GBM 2pta DMG 7 pts Astrocytoma 7 pts Glioma	SD: 50% PR: 2 pts CR: 2 pts mOS: 7.7 mo (GBM); 10.2 mo (GBM ICT and ICV)	No dose-limiting toxicity 2 pts with grade 3 neurologic events
Vitanza et al. 2024 [39]	NCT 04185038 Phase I Brain Child 03- Arm C	B7-H3	ICV infusions of 10 × 10^7^ B7-H3 CAR-T cells Multiple infusions (median 7)	21 pediatric DIPG	mOS for pts after progression: 9.7 mo, before progression: 16.9 mo 3 pts alive 3 yrs from diagnosis	No dose-limiting toxicity Common neurologic events: headache, nausea, vomiting, fever
Zhang et al. 2024 [40]	NCT 05241392 Phase I	B7-H3	ICT or ICV infusions of B7-H3 CAR-T cells 3 × 10^7^ cells (3 pts) 6 × 10^7^ cells (4 pts) 15 × 10^7^ cells (6 pts)	13 adult R/R GBM patients	At 12 mo: 83% OS mOS: 20.3 mo 1 pt: PR 1 pt: CR	No dose-limiting toxicity 2 pts neurologic events gr.3 some pts CRS gr.2
